# Measuring the Performance of Vaccination Programs Using Cross-Sectional Surveys: A Likelihood Framework and Retrospective Analysis

**DOI:** 10.1371/journal.pmed.1001110

**Published:** 2011-10-25

**Authors:** Justin Lessler, C. Jessica E. Metcalf, Rebecca F. Grais, Francisco J. Luquero, Derek A. T. Cummings, Bryan T. Grenfell

**Affiliations:** 1 Department of Epidemiology, Johns Hopkins Bloomberg School of Public Health, Baltimore, Maryland, United States of America; 2 Department of Zoology, University of Oxford, Oxford, United Kingdom; 3 Epicentre, Paris, France; 4 Harvard Humanitarian Initiative, Harvard University, Cambridge, Massachusetts, United States of America; 5 Department of Ecology and Evolutionary Biology, Princeton University, Princeton, New Jersey, United States of America; 6 Forgarty International Center, National Institute of Health, Maryland, United States of America; National Institute for Public Health and the Environment, Netherlands

## Abstract

**Background:**

The performance of routine and supplemental immunization activities is usually measured by the administrative method: dividing the number of doses distributed by the size of the target population. This method leads to coverage estimates that are sometimes impossible (e.g., vaccination of 102% of the target population), and are generally inconsistent with the proportion found to be vaccinated in Demographic and Health Surveys (DHS). We describe a method that estimates the fraction of the population accessible to vaccination activities, as well as within-campaign inefficiencies, thus providing a consistent estimate of vaccination coverage.

**Methods and Findings:**

We developed a likelihood framework for estimating the effective coverage of vaccination programs using cross-sectional surveys of vaccine coverage combined with administrative data. We applied our method to measles vaccination in three African countries: Ghana, Madagascar, and Sierra Leone, using data from each country's most recent DHS survey and administrative coverage data reported to the World Health Organization. We estimate that 93% (95% CI: 91, 94) of the population in Ghana was ever covered by any measles vaccination activity, 77% (95% CI: 78, 81) in Madagascar, and 69% (95% CI: 67, 70) in Sierra Leone. “Within-activity” inefficiencies were estimated to be low in Ghana, and higher in Sierra Leone and Madagascar. Our model successfully fits age-specific vaccination coverage levels seen in DHS data, which differ markedly from those predicted by naïve extrapolation from country-reported and World Health Organization–adjusted vaccination coverage.

**Conclusions:**

Combining administrative data with survey data substantially improves estimates of vaccination coverage. Estimates of the inefficiency of past vaccination activities and the proportion not covered by any activity allow us to more accurately predict the results of future activities and provide insight into the ways in which vaccination programs are failing to meet their goals.

*Please see later in the article for the Editors' Summary*

## Abstract 2

Justin Lessler and colleagues describe a method that estimates the fraction of a population accessible to vaccination activities, and they apply it to measles vaccination in three African countries: Ghana, Madagascar, and Sierra Leone.

## Editors' Summary

**Background:**

Immunization (vaccination) is a proven, cost-effective tool for controlling life-threatening infectious diseases. It provides protection against infectious diseases by priming the human immune system to respond quickly and efficiently to bacteria, viruses, and other disease-causing organisms (pathogens). Whenever the human body is exposed to a pathogen, the immune system—a network of cells, tissues, and organs—mounts an attack against the foreign invader. Importantly, the immune system “learns” from the encounter, and the next time the body is exposed to the same pathogen, the immune system responds much faster to the threat. Immunization exposes the body to a very small amount of a pathogen, thereby safely providing protection against subsequent infection. More than two billion deaths are averted every year through routine childhood immunization and supplemental immunization activities (mass vaccination campaigns designed to increase vaccination coverage where immunization goals have not been reached by routine vaccination). Indeed, these two types of vaccination activities have eliminated smallpox from the world and are close to doing the same for several other infectious diseases.

**Why Was This Study Done?:**

To reduce deaths from infectious diseases even further, it is important to know the proportion of the population reached by vaccination activities. At present, countries report vaccination coverage to the World Health Organization (WHO) that is calculated by dividing the number of vaccine doses delivered during the activity by the size of the target population. However, estimates arrived at through this “administrative method” do not account for vaccine doses that were not actually delivered, and can only reflect a single vaccination activity, which prevents us from identifying populations that may be systematically missed by all vaccination activities (for example, children living in remote areas, or children whose parents refuse vaccination). Moreover, estimates of coverage obtained by the administrative method rarely agree with estimates obtained through cross-sectional surveys such as Demographic and Health Surveys (DHS), which are household surveys of family circumstances and health undertaken at a single time point. In this study, the researchers developed a method for measuring the performance of vaccination activities that estimates the fraction of the population accessible to these activities and within-activity inefficiencies. They then tested their method by applying it to measles vaccination in three African countries; before 1980, measles killed about 2.6 million children worldwide every year, but vaccination activities have reduced this death toll to about 164,000 per year.

**What Did the Researchers Do and Find?:**

The researchers developed a set of formulae (a “likelihood framework”) to estimate the effective coverage of vaccination activities using data on vaccine coverage from cross-sectional surveys and administrative data. They then applied their method to measles vaccination in Ghana, Madagascar, and Sierra Leone using data obtained in each country's most recent DHS survey and administrative data reported to WHO. The researchers estimate that 93%, 77%, and 65% of the target populations in Ghana, Madagascar, and Sierra Leone, respectively, were ever covered by any vaccination activity, and that inefficiencies within vaccination activities were low for Ghana, but higher for Madagascar and Sierra Leone. Consequently, the researchers' estimates of vaccination activity coverage were substantially lower than the administrative estimates for Madagascar and Sierra Leone but only slightly lower than that for Ghana. Finally, the researchers' estimates of routine vaccination coverage were generally lower than WHO-adjusted estimates but broadly agreed with age-specific vaccination coverage levels from DHS surveys.

**What Do These Findings Mean?:**

Although the accuracy of the estimates provided by this likelihood framework depends on the assumptions included in the framework and the quality of the data fed into it, these findings show that, by combining administrative data with survey data, estimates of vaccine coverage can be substantially improved. By providing estimates of both the inefficiency of past vaccination activities and the proportion of the target population inaccessible to any vaccination activity, this method should help public health experts predict the results of future activities and help them understand why some vaccination programs fail to meet their goals. Importantly, knowing both the size of the inaccessible population and the inefficiency level of past programs makes it possible to estimate the effect of providing additional doses of vaccine on vaccination coverage. Finally, the application of this new method might help individual countries understand how susceptibility to specific infectious diseases is building up in their population and enable them to avoid outbreaks similar to the measles outbreaks that have recently occurred in several African countries.

**Additional Information:**

Please access these Web sites via the online version of this summary at http://dx.doi.org/10.1371/journal.pmed.1001080.

- WHO provides information about immunization and details of its Expanded Program on Immunization and its Global Immunization Vision and Strategy; WHO Africa provides details about measles immunization in Africa; a photo story about mass measles vaccination in Côte d’Ivoire is available (some material in several languages)
- The UK National Health Service Choices website provides information for members of the public about immunization
- The Measles Initiative is a collaborative effort that aims to reduce global measles mortality through mass vaccination campaigns and by strengthening routine immunization; its website includes information on measles and measles vaccination, including photos and videos of vaccination activities
- MedlinePlus provides links to additional resources about immunization and about measles (in English and Spanish)
- The charity website Healthtalkonline has interviews with UK parents about their experience of immunizing their children

## Introduction

Immunization is a proven and cost-effective tool for control of infectious disease. Two main types of immunization activities are used to deliver vaccinations to populations, routine immunization and mass campaigns such as Supplemental Immunization Activities (SIAs). Routine immunization occurs year-round, with the aim of providing coverage for all children, as part of the World Health Organization (WHO) Expanded Program on Immunization. In contexts where immunization goals are not met by routine activities, such as measles vaccination in sub-Saharan Africa, SIAs are used to increase vaccination coverage and provide the opportunity for a second dose of vaccine. SIAs occur via campaigns at intervals generally greater than 2 y, targeting a broader range of ages. Throughout this paper, we use the term “immunization activities” to refer to both types of vaccination efforts, and specifically refer to the former as “routine vaccination” and the latter as “campaigns.”

Establishing coverage attained via these immunization activities (i.e., routine coverage and campaigns) is of clear programmatic importance. The coverage of vaccination activities is usually determined by comparing the number of doses distributed during the activity by the size of the target population (the administrative method) [1]. This calculation ignores vaccine wastage and failure to vaccinate inaccessible populations [2], and can sometimes lead to nonsensical results such as “we vaccinated 120% of children from 9 to 48 months of age” [1]. A more direct approach to assessing the success of a vaccination activity is to quantify outcomes (i.e., degree of coverage attained), rather than inputs (i.e., number of vaccines distributed). One measure of outcomes is provided by Demographic and Health Surveys (DHS), nationally representative household surveys undertaken globally and geared to provide comparable data for a wide range of monitoring and impact evaluation indicators for population health, including immunization status [3].

Considering vaccination outcomes (e.g., age-specific vaccination rates) yields more accurate measures of coverage [2], and may also allow identification of key correlates of vaccination (e.g., rural versus urban) [4]. However, considering outcomes alone cannot reveal whether poor coverage is predominantly due to a proportion of the population being inaccessible to vaccination, or predominantly due to distribution inefficiencies and wastage within campaigns and routine activities. Here we show how linking the input information (doses distributed) to the outcomes (age-specific vaccination coverage) allows us to quantify the relative importance of these two components, improving our operational understanding of vaccination activities.

The “inaccessible population” includes those who refuse vaccination (perhaps accounting for the majority of the “unreachable” group in highly developed nations [5]) and those who do not have physical access to vaccination, e.g., people living in remote areas with little access to health care services [6]. In addition to groups who are literally inaccessible, the inaccessible population includes individuals not covered because of overlaps between vaccination activities larger than would be expected by chance alone (i.e., correlations in coverage). For example, overlaps may occur if vaccination activities tend to reach some sub-populations more effectively than others. Hence, while particular immunization activities may have covered more or less of the accessible population, the size of the accessible population represents an upper limit on both the coverage attained by any particular activity and the coverage of all activities combined.

Individuals who are, in theory, targeted and reachable by vaccination activities may also be missed because of inefficiencies within immunization activities, such that not all nominally distributed doses (i.e., doses reported as distributed on country reports) result in an actual new vaccinee. Vaccine wastage may result from discarded doses (due to cold chain lapses or partial use of open vials), vaccination of individuals outside the target population, or revaccination of children already vaccinated within that activity [7]. Note that we consider revaccination an inefficiency only if a child receives two doses in the same activity (e.g., within the same campaign), not if they are vaccinated multiple times in separate activities (e.g., receiving one routine dose and one campaign dose), which may often be desirable. These within-activity inefficiencies dictate how many new vaccinees will be gained for each new dose of vaccine added to a single vaccination activity.

Here we introduce a likelihood formulation that can be used to estimate both the size of the population inaccessible to vaccination activities and the inefficiency in the distribution of vaccine within activities. Taken together, these two quantities dictate both the rate and upper limit of improvement achievable solely by introducing new doses of vaccine into a health system. The analysis provides a method to predict the performance of past vaccination activities and future activities if no systematic changes are made. Also, it may provide some insight into where the vaccine distribution system is failing (e.g., is there a large inaccessible population, or are large numbers of doses being wasted within activities?). Our framework requires only data from a cross-sectional survey measuring age and vaccination status, and information on vaccination activity timing, age range, and number of doses deployed in the years preceding the survey. We illustrate our technique using publicly available data on measles vaccination in Ghana, Madagascar, and Sierra Leone.

## Methods

### Estimating Effective Vaccine Coverage

#### Data requirements

Two sources of data are needed to estimate effective vaccine coverage using our method. The first is administrative data on vaccination activities conducted during the period of interest, including both campaigns and routine vaccination. For each vaccination activity we need to know when the activity occurred, the target age range for the activity, the number of vaccine doses nominally distributed, and the size of the target population. Routine vaccination activities occur over a broad time frame, hence must be treated differently than campaigns in statistical procedures (see “Modeling Routine Vaccination” below). However, from a data standpoint, each year's routine vaccination activities can be represented as a pseudo-campaign occurring on January 1 of that year covering all ages. The first four columns of Table 1 illustrate the data on vaccination activities required by our approach.

**Table 1:** Timing, administrative coverage, and estimated coverage of routine and supplemental vaccination activities for Ghana, Madagascar, and Sierra Leone.

Date	Type	Doses, *V*	Target Population, *N*	Administrative Coverage (100*V*/*N*)	WHO Estimated Coverage	Model Estimated Coverage (95% CI)
**Ghana**						
2003	Routine	646,166	775,191	83%	80%	82% (81, 82)
2004	Routine	660,776	793,461	83%	83%	82% (81, 82)
2005	Routine	718,589	812,221	88%	83%	87% (85, 87)
2006 (Nov 1)	SIA, children aged 9–60 mo	3,994,052	5,065,661	79%	—	77% (77, 78)
2006	Routine	759,222	891,586	85%	85%	83% (82, 84)
2007	Routine	812,083	857,899	95%	95%	92% (91, 93)
2008	Routine	815,617	882,953	92%	86%	90% (89, 91)
**Madagascar**						
2003	Routine	500,960	583,339	86%	56%	67% (65, 69)
2004	Routine	590,167	601,428	98%	58%	72% (70, 75)
2004 (Sep 13)	SIA, children aged 9–168 mo	7,546,229	7,626,090	99%	—	73% (70, 75)
2005	Routine	499,119	595,349	84%	61%	66% (64, 68)
2006	Routine	513,868	612,018	84%	65%	66% (64, 68)
2007	Routine	614,825	629,154	98%	81%	72% (70, 74)
2007 (Oct 22)	SIA, children aged 9–60 mo	3,053,702	3,123,163	98%	—	72% (70, 74)
2008	Routine	620,985	682,680	91%	70%	69% (67, 71)
2009	Routine	595,514	701,795	85%	64%	66% (64, 68)
**Sierra Leone**						
2003	Routine	160,094	185,150	86%	73%	64% (62, 66)
2003 (Oct 28)	SIA, children aged 9–168 mo	2,404,882	2,599,098	93%	—	66% (64, 68)
2004	Routine	139,571	217,438	64%	76%	53% (51, 54)
2005	Routine	153,184	190,143	81%	71%	62% (60, 63)
2006 (Nov 26)	SIA, children aged 9–60 mo	796,509	792,401	101%	—	68% (66, 70)
2006	Routine	155,408	171,908	90%	65%	66% (64, 67)
2007	Routine	155,933	189,149	82%	60%	63% (61, 64)
2008	Routine	190,048	198,251	96%	66%	67% (65, 69)

The second type of data required is a cross-sectional survey of age and vaccination status in the population. This survey may be an age-stratified survey aimed specifically at this question, or any cross-sectional survey where the vaccination status of children of differing ages is obtained (e.g., a DHS survey). Data from vaccination cards, indicating a child's age at the time of routine vaccination, are not necessary but can be used to improve estimates of the age distribution of routine coverage.

#### Vaccination probability and coverage

Suppose that an individual has been in the target population for vaccination activities *V*_1_, *V*_2_,…, *V_m_*. The probability that this individual has been vaccinated is one minus the probability that they avoid vaccination in every activity:
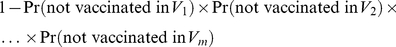
(1)


Let us assume that there exists some population of size 1−ρ that never has a chance of being vaccinated in any activity, which we term the inaccessible population. The probability that an individual is vaccinated is then:
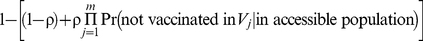
(2)


The probability that an individual in the accessible population is vaccinated during activity *V_j_* is some function of the number of vaccine doses nominally distributed during that activity, *v_j_*, the size of the population targeted by that activity, *N_j_*, and the proportion of that targeted population that is accessible to vaccination activities in general, ρ. If vaccine doses were distributed with perfect efficiency, then this value would be *v_j_*/ρ*N_j_*. However, it seems reasonable to assume that there is some inefficiency in the distribution of vaccine to the accessible target population, and that this inefficiency has a larger effect as more nominal vaccinations occur during an activity. That is, the first nominally distributed dose will nearly assuredly result in an additional vaccinee, but the 1,000th nominally distributed dose has a smaller chance of resulting in an additional vaccinee, and the 100,000th nominally distributed dose has a still smaller chance. We denote this inefficiency ψ, where ψ = 0 denotes an activity with perfect efficiency, i.e., every dose results in an additional vaccinee, and ψ = 1denotes a campaign that is effectively at random, i.e., your chance of receiving any vaccine dose is independent of your chance of receiving a dose previously in that activity (though unlikely, values of ψ>1 are possible, and represent activities worse than at random). Hence, the probability of an individual in the accessible target population remaining unvaccinated during activity *V_j_* is *f*(*v_j_*, ρ*N_j_*, ψ) where (see Text S1 for derivation):
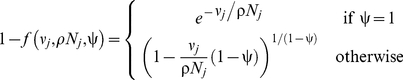
(3)


We can now formalize and simplify Equation 2 above to an expression of the probability that any individual 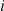 has been vaccinated:
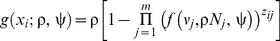
(4)Where *V*_1_, *V*_2_,…, *V_m_* now denotes all vaccination activities that anyone in the population has been exposed to, and *z_ij_* is an indicator of whether person *i* was in the target population for vaccine activity *j*, which is fully determined by the child's age (*x_i_*).

The coverage of a particular vaccination activity, *c_j_*, is the same as the probability that an individual who is in the target population for only that activity (i.e., *z_ij_* = 1 and *z_ik_* = 0 for all *k*≠*j*) is vaccinated. Hence, the expected coverage of activity *j* is:
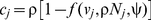
(5)


**Figure 1:**
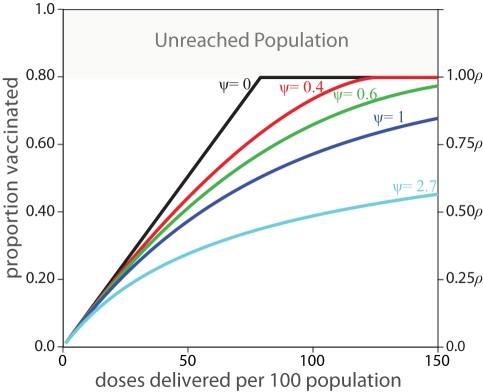
Expected coverage of a vaccination activity based on the values of ψ and ρ. The left *y*-axis presents the proportion of the total target population, while the right *y*-axis shows the proportion vaccinated as a proportion of the accessible population, ρ. A value of ψ = 0 indicates “perfect” vaccine distribution, where every dose of vaccine reaches an individual not yet vaccinated in this activity, ψ = 1 indicates vaccine distribution equivalent to “vaccination at random,” and values of ψ greater than one indicate even worse performance.

**Figure 2:**
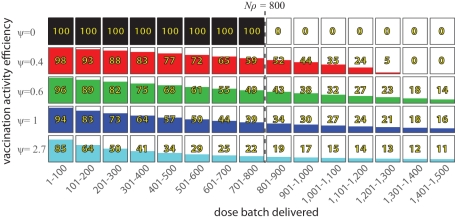
The expected number of additional people vaccinated in a vaccination activity per batch of 100 vaccine doses delivered, in a hypothetical population of 1,000 individuals where ρ = 0.8. If ψ = 0, then each vaccine dose reaches an individual not yet covered by this activity until the entire accessible population is vaccinated (800 individuals in this example). For higher values of ψ, each additional dose delivered into the system has a reduced chance of reaching an individual not yet covered by this activity.

Figures 1 and 2 illustrate how ρ and ψ affect actual coverage. The accessible population, ρ, represents the upper limit of coverage, while the efficiency parameter, ψ, dictates the expected improvement in coverage from the introduction of additional vaccine doses.

#### Modeling routine vaccination

In the simplest formulation, routine vaccination activities can be treated as a single pseudo-campaign occurring when a child reaches the age of recommended vaccination (9 mo for measles), with the routine vaccination coverage reported during the year of that event. However, this approach ignores the fact that different children receive their first dose of vaccine from routine immunization activities at different ages. We can account for this by creating a series of pseudo-campaigns representing the routine vaccination coverage of each year when child *i* was alive. By weighting each pseudo-campaign by the probability of a child having the “opportunity” to be vaccinated in a year's routine activities (*w_ij_*), these pseudo-campaigns can be combined to obtain a child's probability of routine vaccination in their lifetime up to that point (see Text S1):
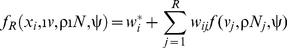
(6)where *j* = 1…*R* are the years of routine vaccination activities and 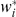 represents the probability that the opportunity for routine vaccination occurs after a child's current age. These weights are calculated as (see Text S1):
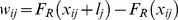
(7)

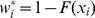
(8)where *F_R_*(*x*) is the probability of having the opportunity for vaccination by age *x*, *x_ij_* is child *i*'s age at the beginning of routine vaccination year *j*, and *l_j_* is the length of exposure to a year's routine activities (12 mo for most years, but truncated in the year of the survey). This new combination of pseudo-campaigns can be included in Equation 4 above as a single vaccination activity.

Sophisticated distributions and forms of estimation for *F_R_*(*x*) are possible, but here we make the simplifying assumption that there is a constant “hazard” λ of routine vaccination after 8.5 mo of age and estimate this hazard in conjunction with the other model parameters. That is:
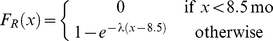
(9)


#### Estimation

In a cross-sectional survey we observe a set of individuals with ages ***x*** = {*x*_1_, *x*_2_,…, *x_n_*} and a set of corresponding vaccination statuses, ***y*** = {*y*_1_, *y*_2_,…, *y_n_*}, where *y_i_* = 0 indicates that an individual has never been vaccinated, and *y_i_* = 1 indicates that he has. Using the formulation from Equation 4, the probability of observing *y_i_* = 1 is *g*(*x_i_*, ρ, ψ), and the probability of observing *y_i_* = 0 is 1−*g*(*x_i_*, ρ, ψ). Assuming that *y*_1_, *y*_2_,…, *y_n_* are independent stochastic variables, the likelihood of the parameters ρ and ψ given these observations can be expressed as the product of the probability of each observation:
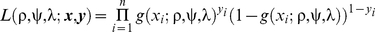
(10)Numeric optimization (e.g., Nelder-Mead) or Markov chain Monte Carlo (MCMC) methods can be used to estimate these parameters.

We extend Equation 10 to use data on the age at time of vaccination for those with vaccine cards to better fit λ, optimizing:
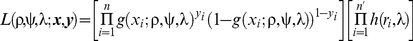
(11)where *n*′ is the number of children with a vaccination card, *r_i_* is the age of routine vaccination on that card, and *h*(*r_i_*, λ) is the probability distribution function for Equation 9. This formulation assumes that the age of routine vaccination is independent of the probability of vaccination given that a child has a vaccination card and that vaccinations recorded on vaccination cards only represent routine vaccination.

### Application to Measles Vaccination

We used data from WHO and country DHS to estimate the effectiveness of measles vaccination activities in Ghana, Madagascar, and Sierra Leone. Countries were selected with the criteria that at least one campaign (i.e., an SIA) occurred within the 5 y prior to the most recent DHS survey, that no campaign occurred in the same year as the most recent DHS survey, and that countries with differing reported vaccination coverage were represented.

Values for the number of doses nominally administered during routine vaccination and the size of the target population are based on the values reported by countries to WHO (data provided by WHO). WHO–United Nations Children's Fund (UNICEF) estimates of national immunization coverage for comparison were obtained from WHO [8]. Information on when campaigns occurred, doses deployed during campaigns, and the age range targeted were obtained from WHO.

Measles vaccination status for children from 9 to 59 mo of age was obtained from country-specific DHS surveys [9]–[11]. Children were considered to have been vaccinated for measles if vaccination was recorded on the child's health card or the child's mother reported that the child had been vaccinated (Table 2). Vaccination status, age, and timing of interview were obtained for 2,304 children from Ghana (DHS survey in 2008), *n* = 9,747 for Madagascar (DHS survey in 2008–2009), and *n* = 3,966 for Sierra Leone (DHS survey in 2008). Age at time of routine vaccination was calculated for those children where the DHS data indicated a vaccination card had been seen and a date of vaccination was recorded (*n* = 1,550 for Ghana, 3,787 for Madagascar, and 1,216 for Sierra Leone).

**Table 2:** Details of DHS data, contrasting numbers, and percent reported vaccinated by age as ascertained by vaccination cards, as reported by mothers, and from either source.

Country	Year	Number Surveyed	Vaccination Report Source	Age			
				9–23 mo	24–35 mo	36–47 mo	48–60 mo
**Ghana**	2008	2,304	Card	529 (72%)	388 (76%)	307 (61%)	326 (58%)
			Mother	70 (10%)	89 (18%)	136 (27%)	185 (33%)
			Either	599 (82%)	477 (94%)	443 (88%)	511 (91%)
			Total surveyed	734	508	501	561
**Madagascar**	2008–2009	9,747	Card	1,147 (42%)	984 (41%)	845 (38%)	811 (33%)
			Mother	539 (20%)	815 (34%)	846 (38%)	1,037 (43%)
			Either	1,686 (62%)	1,799 (76%)	1,691 (75%)	1,848 (77%)
			Total surveyed	2,724	2,374	2,250	2,399
**Sierra Leone**	2008	3,966	Card	454 (35%)	322 (36%)	229 (25%)	211 (25%)
			Mother	272 (21%)	286 (32%)	393 (42%)	366 (43%)
			Either	726 (56%)	608 (68%)	622 (67%)	577 (68%)
			Total surveyed	1,302	895	925	844

In some cases a card was present, but the date of vaccination was not recorded.

Parameter estimates and 95% confidence intervals were obtained using MCMC methods (the Metropolis-Hasting algorithm). All MCMC chains were started from ρ = 0.5, ψ = 1, and λ = 1. Model convergence was checked by examination of MCMC chains, comparison of the posterior distributions estimated from different chains, calculation of 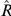 [12], and comparison with results from numerical fitting procedures (Nelder-Mead). Five chains of length 5,000 were run for each country, and the posterior distribution was estimated to be the empirical distribution of the combined set of the last 2,500 iterations from all chains. Reported parameter estimates are the medians of the posterior distributions (posteriors were normally distributed on the scale used in estimation), and 95% confidence intervals from quantiles of the posterior distribution. The estimated coverage for each campaign and confidence intervals were similarly obtained from the posterior distribution created by applying Equation 5.

Confidence intervals on the age distribution of vaccination reported in DHS data were obtained by bootstrapping (500 iterations). Confidence intervals for model estimates of the age distribution of vaccination were obtained by performing 500 parametric bootstrap iterations where (a) parameters were sampled from the posterior distribution, (b) a bootstrap population is created based on the DHS data, and (c) each individual in the bootstrap population is randomly assigned a vaccination status based on the parameters selected in step a. This procedure accounts for (a) uncertainty in parameter estimates, (b) uncertainty in population structure, and (c) uncertainty from the Bernoulli process; confidence intervals are thus comparable with those obtained from the DHS data.

The age distribution that would have resulted from naïve use of WHO-corrected estimates of coverage was calculated assuming independence between vaccination activities and routine vaccination at 9 mo of age. Campaigns were assumed to have coverage that differed from that reported by the same proportion as the routine vaccination activities occurring that year. Confidence intervals were calculated using steps b and c above.

All statistical analyses were done in the R statistical package, version 2.11 (http://cran.r-project.org).

## Results

We estimate that 7% (95% CI: 6, 9) of children in Ghana, 23% (95% CI: 24, 22) of children in Madagascar, and 31% (95% CI: 33, 30) of children in Sierra Leone were never accessible by routine measles vaccination or campaigns (Table 3). The estimated inefficiency within vaccination activities was highest in Madagascar (ψ = 0.34, 95% CI: 0.28, 0.41), followed by Sierra Leone (ψ = 0.33, 95% CI: 0.31, 0.39), then Ghana (ψ = 0.03, 95% CI: 0.02, 0.04). Hence, our estimates of routine and SIA coverage are substantially lower than administrative estimates for Madagascar and Sierra Leone, and only slightly lower for Ghana (Table 1). Our estimates of routine coverage are in general lower than the WHO-UNICEF estimates generated by a heuristic method.

**Table 3:** Estimated proportion of the population accessible by any vaccination activity (ρ), within-activity inefficiency (ψ), and routine vaccination opportunity rate (λ) for Ghana, Madagascar, and Sierra Leone.

Country	ρ	ψ	λ
**Ghana**	93% (95% CI: 91, 94)	0.03 (95% CI: 0.02, 0.04)	0.66 (95% CI: 0.54, 0.82)
**Madagascar**	77% (95% CI: 76, 78)	0.34 (95% CI: 0.28, 0.41)	0.60 (95% CI: 0.49, 0.73)
**Sierra Leone**	69% (95% CI: 67, 70)	0.33 (95% CI: 0.31, 0.39)	0.47 (95% CI: 0.38, 0.61)

Based on our estimated routine vaccination distribution (λ; Table 3), children in all three countries who receive routine vaccination do so near their 9-mo birthday. In Ghana, children receive routine vaccination at a slightly younger age (mean age = 10.0 mo) than in Madagascar (mean age = 10.2 mo) or Sierra Leone (mean age = 10.6 mo). These estimates are slightly lower than estimates obtained from the empirical distribution of ages reported on vaccination cards (mean = 10.4, 10.8, and 12.2 mo respectively), reflecting that the constant rate assumption is not precisely correct.

**Figure 3:**
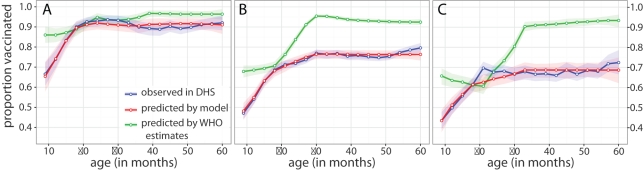
DHS and model estimates of age-specific vaccination coverage. Proportion vaccinated by age in (A) Ghana, (B) Madagascar, and (C) Sierra Leone. Points connected by solid lines are the window-smoothed (age ±5 mo) estimates of vaccine coverage as measured by the most recent DHS survey (blue), as predicted by naïve application of WHO coverage estimates (green), and as fit using our approach (red) in a population with the same age distribution as the DHS data. Shaded regions represent 95% confidence intervals on the window-smoothed estimate calculated as described in Methods.

A comparison of the age-specific proportion vaccinated predicted by our approach with the proportion vaccinated observed in the DHS data shows substantial agreement (Figure 3). For Madagascar and Sierra Leone, our predictions show substantially better agreement with the DHS data than what is predicted by naïve application of WHO-adjusted coverage estimates. For Ghana, where vaccination coverage is relatively high, the unreachable population is small, and wastage very low, projections from WHO generally agree with our estimates and the proportion vaccinated observed in the DHS.

With the exception of Ghana, we find that the models including both ρ and ψ as free parameters model the data substantially better, as measured by Akaike information criterion, than models where we assume a fully accessible population (ρ = 1) or perfect efficiency (ψ = 0) (Table S1). In the case of Ghana, where ψ is estimated to be nearly zero, assuming perfect efficiency leads to a slightly improved but qualitatively equivalent model of the DHS data.

## Discussion

Reliable estimates of vaccination coverage are key to managing population immunization status. Estimates of coverage are usually based on administrative measures (i.e., the ratio of distributed doses to the size of the target population). Refinements of this approach do exist; however, current WHO adjustments of estimates of coverage employ a heuristic method in which “no statistical or mathematical models are used.” [1]. Data quality audits [13] provide a verification factor that as yet has not proved sufficiently stable to be useful [14]. Here we have introduced a method by which administrative coverage estimates can be combined with a cross-sectional survey to estimate the effectiveness of vaccination programs. This method not only attempts to correct coverage estimates, but also distinguishes between issues of overall coverage and vaccine wastage within vaccination activities.

The causes of variation between country-specific levels of inefficiency and overlap between campaigns (Table 3) may be diverse, but general conclusions about immunization activities emerge. While a combination of routine immunization via the WHO Expanded Program on Immunization and campaigns can successfully maintain high coverage in countries like Ghana, the analysis shows how in contexts like that of post-conflict Sierra Leone, or Madagascar, the strategy itself may be inadequate. For example, in Sierra Leone, despite investment in two campaigns in 3 y, both these campaigns and the underlying routine program failed to reach 30% of children; and in 2010, there was a large outbreak in children aged greater than 5 y in Freetown. More generally, wherever such a large proportion of the population remains inaccessible, e.g., when there is high overlap between campaigns, the combined strategy of vaccination via the Expanded Program on Immunization and via SIAs is unlikely to succeed, and refocusing effort into the design of vaccination strategy (e.g., improving the vaccine distribution structure and finding novel ways to target unreached populations) should yield considerable rewards in terms of improved coverage.

For our technique to be useful, countries must have cross-sectional data on vaccine coverage for children across a range of ages, some of an age where they have been exposed to multiple vaccination activities (e.g., one or more campaigns and routine vaccination). As illustrated here, a DHS survey provides sufficient information, but surveys aimed specifically at measuring coverage that target a wider age range (ideally paired with serosurveys) could improve estimates. Once countries have estimates of the accessible population and within-campaign inefficiencies, they can predict the coverage of activities occurring after the cross-sectional survey (using Equation 5) and the age-specific coverage obtained after these activities (using Equation 4). With these estimates in hand, countries can better understand how susceptibility may be building up in their population, perhaps enabling them to avoid outbreaks of the type and scale observed recently in Sierra Leone, Malawi, Zambia, and South Africa.

It may seem surprising that both ψ and ρ are identifiable using only age-stratified vaccination prevalence. However, simulations show that differences in these values lead to significant differences in the age profile of vaccination coverage when children of differing ages have differing exposure to multiple vaccination activities (Figures S1 and S2). If children are not exposed to multiple activities (e.g., there is only routine vaccination), it will not necessarily be possible to distinguish between these two sources of program inefficiency. A critical way estimates could be improved is by conducting cross-sectional surveys of vaccination coverage covering ages greater than 5 y (the current upper limit in DHS surveys). Such surveys would include individuals who had been potentially covered by more vaccination campaigns, improving estimates and allowing for separate analysis of inefficiencies in routine vaccination and SIAs.

Limitations of our approach include required assumptions that may not always be justified. The assumption of constant inefficiencies across the study period may not be appropriate, especially in the context of a global measles elimination campaign. A potential extension to account for this variation would be to allow estimates of ρ and ψ to vary smoothly over time. The assumption that the rate at which children have the opportunity to receive routine vaccination is constant after 8.5 mo of age is clearly an oversimplification. While more sophisticated techniques can be used to fit this age distribution, as in [15], the simplified approach still fits the age distribution seen in the DHS data well (Figure 3).

Our sample estimates are also subject to the quality of the available data. We assume that the target population is accurately estimated. A sensitivity analysis of the effect of over- or underestimates of the target population indicates that such misspecifications do not much bias estimates of the size of the unreachable population, but do impact estimates of within-campaign efficiency (Table S2). Underestimates of the size of the target population lead to underestimates of within-campaign efficiency (i.e., overestimates of ψ), and overestimates lead to overestimates of within-campaign efficiency (i.e., underestimates of ψ). Hence, the high within-campaign inefficiency estimates for Sierra Leone and Madagascar could be partially the result of poorly specified denominators, particularly as in both cases at least some of the immunization activities were performed long after the last census (Table S3).

We also assume that the DHS data are representative of each country. If DHS surveys in fact missed populations that were also missed by immunization activities, the size of the population inaccessible to vaccination would be underestimated. Another potential source of bias is that a high proportion of the vaccination data come from reporting by the mother (Table 2), particularly in Sierra Leone. If mothers report more children have been vaccinated than is in fact the case, both the size of the accessible population and the efficiency of campaigns may be overestimated, and the reverse if mothers report fewer children vaccinated than there really are. Additionally, in all countries, the proportion of reports from the mother increases with child age, reflecting the fact that vaccination cards are more rarely distributed during SIAs. This could lead to either over- or underreporting of vaccination occurring during SIAs. If underreporting of campaign coverage is occurring, the model will tend to both underestimate overall coverage and overestimate within-campaign inefficiency. Conversely, if campaign coverage is overreported, our technique will underestimate within-campaign inefficiencies. Age-specific serosurveys would provide a valuable benchmark by which to evaluate the coverage estimates, and could perhaps be paired with existing research, monitoring, or vaccination activities.

The estimates obtained by our method bear an inconsistent relationship to the WHO-UNICEF adjusted estimates (Table 1). In some places we estimate significantly lower coverage (e.g., Sierra Leone in 2004) and in others we estimate higher coverage (e.g., Ghana in 2005). In all cases our estimates are the result of the reported administrative coverage and the estimated model parameters. Where we estimate a large accessible population and low inefficiency, our estimates will be high relative to administrative coverage; where we estimate a small accessible population and high inefficiency, our estimates will be relatively low. We assume model parameters are constant over the 5 y considered, while the heuristics used in the WHO-UNICEF estimates may capture specific short-term factors of which we are unaware [1], and hence may be more accurate for individual years. However, without further information, it is unclear how to combine yearly WHO-UNICEF estimates to the get full age distribution of vaccine coverage (assuming independence clearly performs poorly; Figure 3). Since the assessment of coverage from multiple activities is integral to our approach, our approach has some clear advantages despite its limitations.

The method presented here provides a way in which the performance of vaccination activities can be more accurately measured (and can be extended to consider, e.g., the problem of access to a second dose; Figure S3). As illustrated by our examples, this approach can be used to produce estimates of effective vaccine coverage that are more consistent with the proportion of the population reporting vaccination than current approaches are. These estimates go beyond mere measures of cross-sectional coverage obtained directly from a DHS survey, characterizing the performance of the activities leading to that coverage, and helping to predict the effect of future vaccination activities. Our estimates can be used to identify those countries where problems in vaccine delivery may exist (e.g., Madagascar and Sierra Leone), thereby providing important operational guidance as to how vaccine coverage may be improved. Such guidance is essential if measles control goals are to be met.

## Supporting Information

Alternative Language Abstract S1 French translation of the abstract by CJEM and FJL.
(DOC)

Figure S1 **Log likelihood surfaces for four parameter combinations.** This figure shows in each case the “true” parameter values (used in the simulation) as a black point and the peak of the log likelihood surface as a red point. Surfaces are based on simulated vaccine outcomes for populations of 4,000 individuals across an age range of 9 to 60 mo, with ρ values of 0.75 (corresponding to a large unreachable population) and 0.95 (a small unreachable population) and ψ values of 9 (high vaccine wastage) and 0 (high efficiency and low wastage). We assumed three SIA campaigns, each occurring a year apart, targeting children aged 9 to 60 mo, and with coverage of 0.65. For clarity, we assumed no routine vaccination.
(TIF)

Figure S2 **Proportion vaccinated over age for four parameter combinations.** This figure demonstrates the benefits achievable by decreasing the size of the unreachable population (i.e., increasing ρ) or decreasing wastage (i.e., decreasing ψ). The figure is based upon simulated vaccine outcomes for 4,000 individuals (see Figure S1).
(TIF)

Figure S3 **Probability of vaccination in multiple activities.** The estimated probability of a child having received two or more doses of measles vaccine by age in the entire population (red) and the accessible population only (orange) in (A) Ghana, (B) Madagascar, and (C) Sierra Leone. Estimates assume an independent probability of receiving a vaccination in each vaccination activity given that an individual is in the accessible population. In population-based estimates (red) the probability of being in the accessible population is considered to be ρ; in accessible population estimates this probability is considered to be one. Confidence intervals are calculated as described in Methods.
(TIF)

Table S1 **Comparison of model fits to DHS data using the full model.** This table compares the performance of the full model to a model where the entire population is assumed to be accessible (ρ = 1) and to a model where campaigns are assumed to have perfect efficiency (ψ = 0). Maximum likelihood estimates of parameters were determined using Nelder-Mead numeric optimization, and in some cases differ slightly (by less than .01) from MCMC-based estimates in the main analysis.
(DOCX)

Table S2 **The effect of misspecified target population size.** (A) Simulation results for the mean bias when estimating ψ and ρ with correct and misspecified target population size. To examine possible bias in the estimation procedure, as well as biases resulting from misspecification of the denominator, we performed 500 simulations of a population of 4,000 individuals and estimated ψ and ρ with the assumed target population size being 80%, 95%, 100%, 105%, and 120% of its true value. We assessed the mean bias in estimates of ψ and ρ (Table S1). We found that estimates of the size of the unreachable population (ρ) were only biased when size of the target population was severely overestimated (120% of its actual value). Estimates of ψ are more sensitive to the specification of the denominator, with underestimates leading to substantial underestimates of within-campaign efficiency (i.e., overestimates of ψ), and overestimates leading to overestimates of within-campaign efficiency (i.e., underestimates of ψ). (B) Campaign coverage biases in percentages corresponding to parameter estimates reflecting mean biases from population misspecification in (A).
(DOCX)

Table S3 **SIAs and population census timing and coverage.** Details of SIAs (years, estimated target population, and estimated doses delivered; from [8]) and data on date of last population census previous to each campaign from [16].
(DOCX)

Text S1 **Mathematical derivations.**
(PDF)

## Abbreviations

DHS: Demographic and Health Surveys
MCMC: Markov chain Monte Carlo
SIA: Supplemental Immunization Activity
UNICEF: United Nations Children's Fund
WHO: World Health Organization
