# Supplementary material for: Measuring the Performance of Vaccination Programs Using Cross-Sectional Surveys: A Likelihood Framework and Retrospective Analysis
Source: PLoS Med. 2011 Oct 25;8(10):e1001110. doi: 10.1371/journal.pmed.1001110 (PMC3201935; doi:10.1371/journal.pmed.1001110)
Supplement: Alternative Language Abstract S1 — French translation of the abstract by CJEM and FJL. (DOC) [file pmed.1001110.s001.doc]

### Background

## The performance of routine and supplemental immunization activities is usually measured by the administrative method: dividing the number of doses distributed by the size of the target population. This method leads to coverage estimates that are sometimes impossible (e.g., vaccination of 102% of the target population), and are generally inconsistent with the proportion found to be vaccinated in Demographic and Health Surveys (DHS). We describe a method that estimates the fraction of the population accessible to vaccination activities, as well as within campaign inefficiencies, thus providing a consistent estimate of vaccination coverage.

### Methods and Findings

We develop a likelihood framework for estimating the effective coverage of vaccination programs using cross-sectional surveys of vaccine coverage combined with administrative data. We applied our method to measles vaccination in three African countries: Ghana, Madagascar and Sierra Leone, using data from each country’s most recent DHS and administrative coverage data reported to the WHO. We estimate that 93% (95% CI: 91, 94) of the target population in Ghana was ever covered by any measles vaccination activity, 77% (95% CI: 78, 81) in Madagascar and 69% (95% CI: 67, 70) in Sierra Leone. Within activity inefficiencies were estimated to be low in Ghana, and higher in Sierra Leone and Madagascar.  Our model successfully fits age specific vaccination coverage levels seen in DHS data, which differ markedly from those predicted by naïve extrapolation from country reported and WHO adjusted vaccination coverage.

### Conclusions

## Combining administrative data with survey data substantially improves estimates of vaccination coverage. Estimates of the inefficiency of past vaccination activities and the proportion not covered by any activity allow us to more accurately predict the results of future activities and provide insight into the ways in which vaccination programs are failing to meet their goals.

**Introduction**

La performance des services de vaccination de routine et des activités de vaccination supplémentaires est normalement évaluée par la méthode administrative, c'est-à-dire en divisant le nombre de doses distribuées par la taille de la population ciblée. Cette méthode conduit à des estimations de couvertures qui sont parfois aberrantes (par exemple une couverture de 102% de la population ciblée), et son généralement incompatibles avec les couvertures vaccinales estimées dans les Enquêtes Démographiques et de Santé (EDS). Nous décrivons ici une méthode qui permet d’estimer la fraction de la population accessible aux activités de vaccination, ainsi que le degré d’inefficacité de la campagne de vaccination, de façon à obtenir une estimation cohérente de la couverture vaccinale.

**Méthodes et Résultats**

Nous avons développé un **système de fonctions de vraisemblance** pour estimer la couverture effective de programmes de vaccination à partir de résultats d’enquêtes transversales de couverture vaccinale combinés avec des données administratives. Nous avons appliqué notre méthode à trois pays africains (Ghana, Sierra Leone, et Madagascar) en utilisant des données du plus récent EDS de chaque pays, et des données de couverture administratives rapportées par l'OMS. Nous avons estimé que 93% (95% CI: 91, 94) de la population ciblée au Ghana a été couverte par l’ensemble des activités de vaccination contre la rougeole, 77% (95% CI: 78, 81) à Madagascar et 69% (95% CI: 67, 70) en Sierra Leone. Le degré d’inefficacité estimé était bas au Ghana, et plus élevé en Sierra Leone et à Madagascar. Notre modèle montrait une bonne concordance avec les couvertures vaccinales par classe d’âge observées dans les enquêtes EDS, qui diffèrent considérablement de celles prédites par une extrapolation naïve des valeurs rapportées par les pays et ajustées par l’OMS.

Translate from: [Spanish](javascript:void(0))Type text or a website address or [translate a document.](http://translate.google.com/?tr=f&hl=en)[Cancel](http://translate.google.com/?tr=t&hl=en)

**English - detected to French translation**

**Conclusion**

La combinaison des données administratives avec les résultats des enquêtes de couverture vaccinale permet d’améliorer sensiblement les estimations de couverture. Les estimations de l'inefficacité des activités de vaccination précédentes et de la proportion de la population qui n’a jamais bénéficié de ces activités permettent de prédire plus précisément les résultats de futures activités et de comprendre les obstacles empêchant les programmes vaccinaux d’atteindre leurs objectifs.
