# Supplementary figures and images for: Measuring the Performance of Vaccination Programs Using Cross-Sectional Surveys: A Likelihood Framework and Retrospective Analysis
Source: PLoS Med. 2011 Oct 25;8(10):e1001110. doi: 10.1371/journal.pmed.1001110 (PMC3201935; doi:10.1371/journal.pmed.1001110)

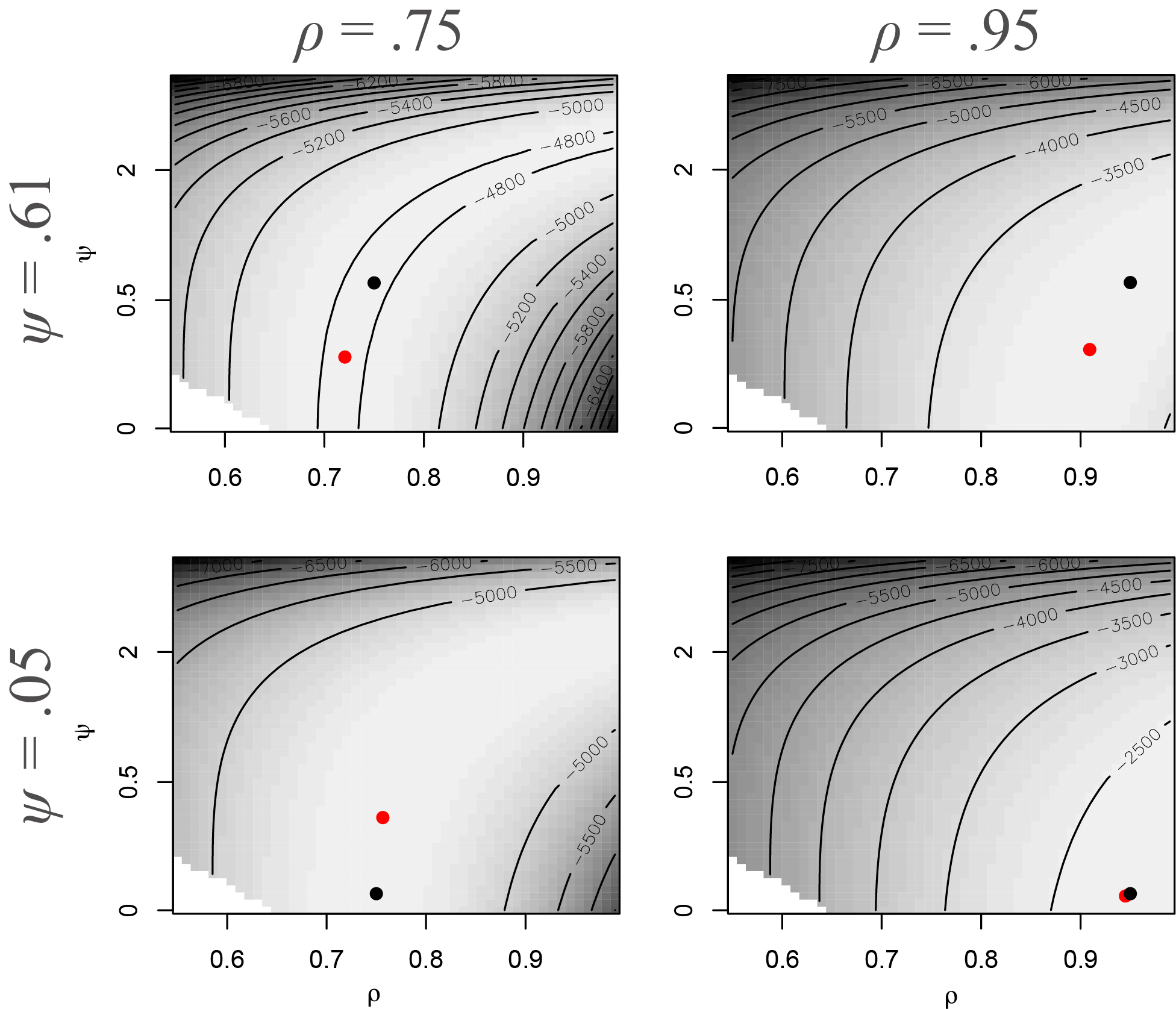

Supplement: Figure S1 — Log likelihood surfaces for four parameter combinations. This figure shows in each case the “true” parameter values (used in the simulation) as a black point and the peak of the log likelihood surface as a red point. Surfaces are based on simulated vaccine outcomes for populations of 4,000 individuals across an age range of 9 to 60 mo, with ρ values of 0.75 (corresponding to a large unreachable population) and 0.95 (a small unreachable population) and ψ values of 9 (high vaccine wastage) and 0 (high efficiency and low wastage). We assumed three SIA campaigns, each occurring a year apart, targeting children aged 9 to 60 mo, and with coverage of 0.65. For clarity, we assumed no routine vaccination. (TIF) [file pmed.1001110.s002.tif]

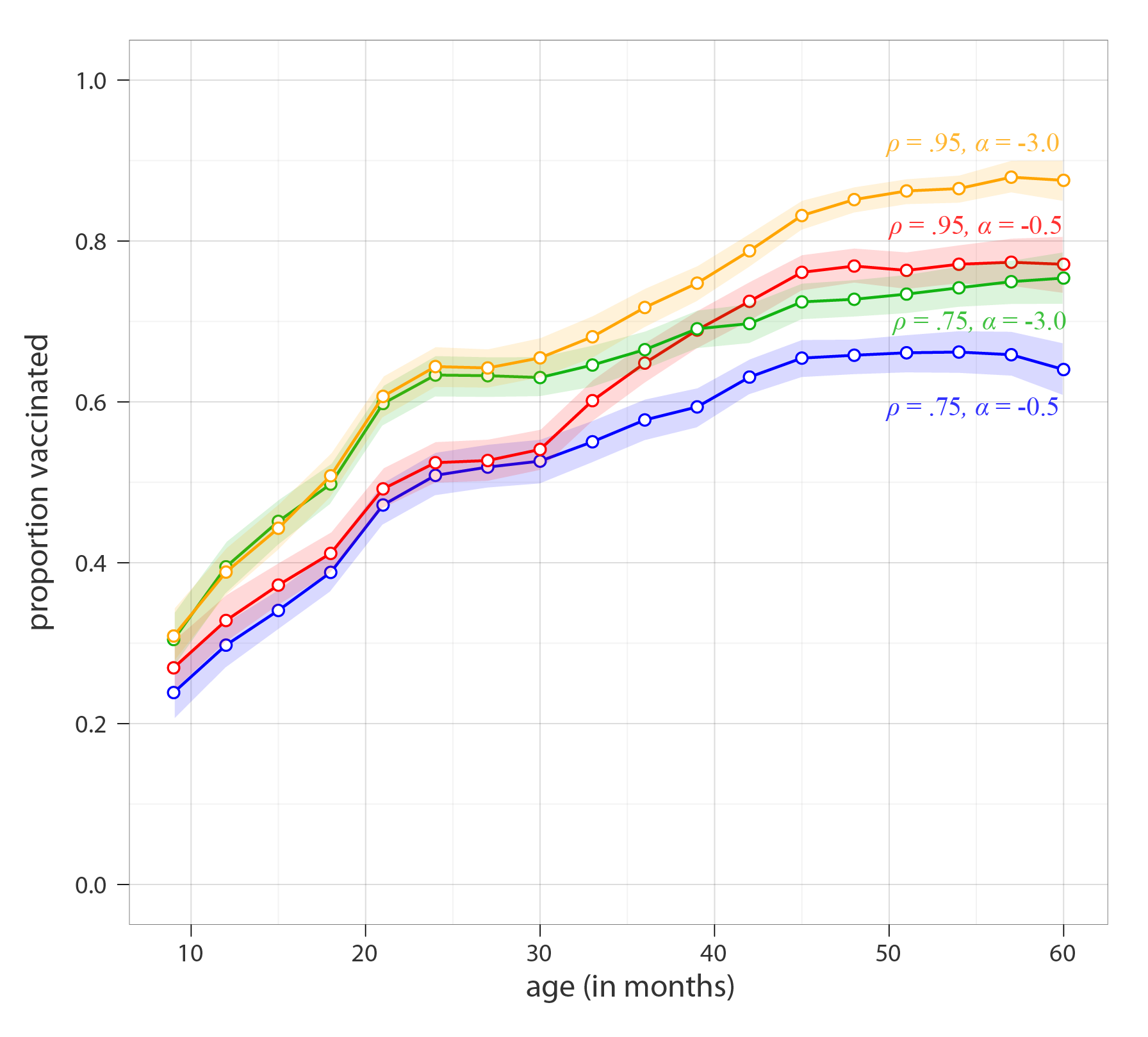

Supplement: Figure S2 — Proportion vaccinated over age for four parameter combinations. This figure demonstrates the benefits achievable by decreasing the size of the unreachable population (i.e., increasing ρ) or decreasing wastage (i.e., decreasing ψ). The figure is based upon simulated vaccine outcomes for 4,000 individuals (see Figure S1). (TIF) [file pmed.1001110.s003.tif]

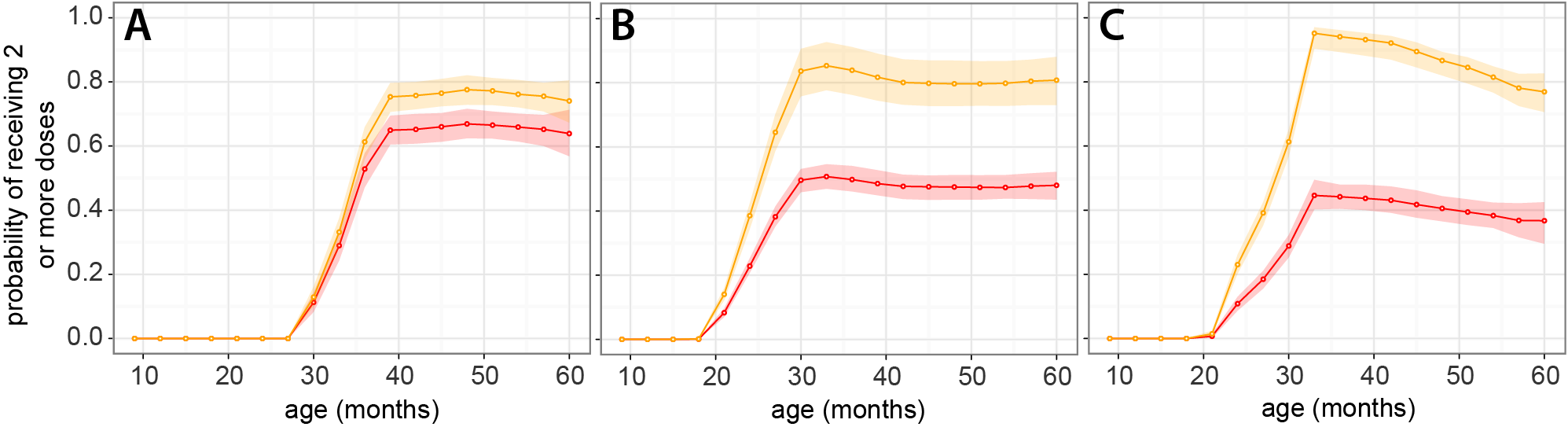

Supplement: Figure S3 — Probability of vaccination in multiple activities. The estimated probability of a child having received two or more doses of measles vaccine by age in the entire population (red) and the accessible population only (orange) in (A) Ghana, (B) Madagascar, and (C) Sierra Leone. Estimates assume an independent probability of receiving a vaccination in each vaccination activity given that an individual is in the accessible population. In population-based estimates (red) the probability of being in the accessible population is considered to be ρ; in accessible population estimates this probability is considered to be one. Confidence intervals are calculated as described in Methods. (TIF) [file pmed.1001110.s004.tif]
