# Supplementary material for: Measuring the Performance of Vaccination Programs Using Cross-Sectional Surveys: A Likelihood Framework and Retrospective Analysis
Source: PLoS Med. 2011 Oct 25;8(10):e1001110. doi: 10.1371/journal.pmed.1001110 (PMC3201935; doi:10.1371/journal.pmed.1001110)
Supplement: Table S1 — Comparison of model fits to DHS data using the full model. This table compares the performance of the full model to a model where the entire population is assumed to be accessible (ρ = 1) and to a model where campaigns are assumed to have perfect efficiency (ψ = 0). Maximum likelihood estimates of parameters were determined using Nelder-Mead numeric optimization, and in some cases differ slightly (by less than .01) from MCMC-based estimates in the main analysis. (DOCX) [file pmed.1001110.s005.docx]

**Table S1:**

|  | $\rho$ | $\psi$ | $\lambda$ | Log Likelihood | AIC |
| --- | --- | --- | --- | --- | --- |
| **Ghana** |  |  |  |  |  |
| **base** | 0.92 | 0.02 | 0.65 | -763 | 1532 |
| **fixed** $\rho$ | 1.00 | 0.11 | 0.77 | -800 | 1604 |
| **fixed** $\psi$ | 0.91 | 0.00 | 0.59 | -762 | 1528 |
| **Madagascar** |  |  |  |  |  |
| **base** | 0.77 | 0.34 | .60 | -5550 | 11107 |
| **fixed** $\rho$ | 1.00 | 1.38 | 1.12 | -5663 | 11330 |
| **fixed** $\psi$ | 0.79 | 0.00 | 0.18 | -5680 | 11366 |
| **Sierra Leone** |  |  |  |  |  |
| **base** | 0.69 | 0.32 | .46 | -2525 | 5055 |
| **fixed** $\rho$ | 1.00 | 1.79 | .98 | -2625 | 5254 |
| **fixed** $\psi$ | 0.72 | 0.00 | .17 | -2548 | 5100 |
