# Supplementary material for: Measuring the Performance of Vaccination Programs Using Cross-Sectional Surveys: A Likelihood Framework and Retrospective Analysis
Source: PLoS Med. 2011 Oct 25;8(10):e1001110. doi: 10.1371/journal.pmed.1001110 (PMC3201935; doi:10.1371/journal.pmed.1001110)
Supplement: Table S2 — The effect of misspecified target population size. (A) Simulation results for the mean bias when estimating ψ and ρ with correct and misspecified target population size. To examine possible bias in the estimation procedure, as well as biases resulting from misspecification of the denominator, we performed 500 simulations of a population of 4,000 individuals and estimated ψ and ρ with the assumed target population size being 80%, 95%, 100%, 105%, and 120% of its true value. We assessed the mean bias in estimates of ψ and ρ (Table S1). We found that estimates of the size of the unreachable population (ρ) were only biased when size of the target population was severely overestimated (120% of its actual value). Estimates of ψ are more sensitive to the specification of the denominator, with underestimates leading to substantial underestimates of within-campaign efficiency (i.e., overestimates of ψ), and overestimates leading to overestimates of within-campaign efficiency (i.e., underestimates of ψ). (B) Campaign coverage biases in percentages corresponding to parameter estimates reflecting mean biases from population misspecification in (A). (DOCX) [file pmed.1001110.s006.docx]

***Table S2A***

| ***true value*** | | ***estimated population size as a percentage of actual population*** | | | | | | | | | |
| --- | --- | --- | --- | --- | --- | --- | --- | --- | --- | --- | --- |
| $\psi$ | $\rho$ | **80%** | | **95%** | | **100%** | | **105%** | | **120%** | |
|  |  | $\psi$ | $\rho$ | $\psi$ | $\rho$ | $\psi$ | $\rho$ | $\psi$ | $\rho$ | $\psi$ | $\rho$ |
| 0.61 | 0.75 | 0.43 | -0.00 | 0.11 | 0.00 | 0.00 | 0.00 | -0.10 | 0.00 | -0.38 | 0.00 |
| 0.61 | 0.95 | 0.58 | 0.00 | 0.15 | 0.00 | 0.00 | 0.00 | -0.13 | -0.00 | -0.50 | 0.00 |
| 0.05 | 0.75 | 0.32 | 0.00 | 0.09 | 0.00 | 0.01 | 0.00 | -0.04 | 0.00 | -0.05 | 0.03 |
| 0.05 | 0.95 | 0.48 | 0.00 | 0.11 | 0.00 | 0.01 | 0.00 | -0.05 | 0.01 | -0.05 | 0.05 |

***Table S2B***

| **true coverage** | **estimated population size as a percentage of actual population** | | | | |
| --- | --- | --- | --- | --- | --- |
|  | **80%** | **95%** | **100%** | **105%** | **120%** |
| 49% | -0.02 | 0.17 | 0.06 | 0.06 | 0.13 |
| 52% | -1.84 | -0.31 | 0.05 | 0.52 | 2.03 |
| 62% | 3.16 | 1.20 | 0.01 | 1.01 | 8.75 |
| 63% | -0.81 | -0.02 | 0.01 | 1.64 | 9.38 |
