# Supplementary material for: Measuring the Performance of Vaccination Programs Using Cross-Sectional Surveys: A Likelihood Framework and Retrospective Analysis
Source: PLoS Med. 2011 Oct 25;8(10):e1001110. doi: 10.1371/journal.pmed.1001110 (PMC3201935; doi:10.1371/journal.pmed.1001110)
Supplement: Table S3 — SIAs and population census timing and coverage. Details of SIAs (years, estimated target population, and estimated doses delivered; from [8]) and data on date of last population census previous to each campaign from [16]. (DOCX) [file pmed.1001110.s007.docx]

***Table S3***

| **Country** | **Year** | **Ages covered (months)** | **Target population** | **Doses delivered** | **Percent reached** | **Year of census previous to SIA** |
| --- | --- | --- | --- | --- | --- | --- |
| Ghana | 2006 | 9-60 | 5,065,661 | 3,994,052 | 79% | 2000 |
|  |  |  |  |  |  |  |
| Madagascar | 2004 | 9-168 | 7,626,090 | 7,546,229 | 99% | 1993 |
|  | 2007 | 9-60 | 3,123,163 | 3,053,702 | 100% | 1993 |
|  |  |  |  |  |  |  |
| Sierra Leone | 2003 | 9-168 | 2,599,098 | 2,404,882 | 92% | 1985 |
|  | 2006 | 9-60 | 748,209 | 751,107 | 100% | 2004 |
