# Supplementary material for: Measuring the Performance of Vaccination Programs Using Cross-Sectional Surveys: A Likelihood Framework and Retrospective Analysis
Source: PLoS Med. 2011 Oct 25;8(10):e1001110. doi: 10.1371/journal.pmed.1001110 (PMC3201935; doi:10.1371/journal.pmed.1001110)
Supplement: Text S1 — Mathematical derivations. (PDF) [file pmed.1001110.s008.pdf]

Supplement to "Measuring the performance of vaccination programs using cross-sectional surveys": Mathematical Derivations

Justin Lessler C. Jessica E. Metcalf Rebecca F. Grais
Francisco J. Luquero Derek A.T. Cummings Bryan T. Grenfell

1 Individual Vaccination Probability and Likelihood Formulation

The probability that an individual is vaccinated at age x is one minus the probability that they avoid vaccination during every vaccination activity to which they are exposed. Assume that there is some portion of the population, ρ, that is accessible to vaccination activities:

$$g(x;\rho) = 1 - \left[(1-\rho) + \rho \prod_{j=1}^{m} \Pr(\text{not vaccinated in } V_j | \text{ accessible})\right]$$

where $V_1, ..., V_m$ are all vaccination activities to which the child might have exposed. Let $f(V_j)$ be the probability of not being vaccinated in activity j given that you are in the target population for that activity and in the accessible population. Let $z_{ij} = 1$ if person i is in the target population for campaign j, and $z_{ij} = 0$ otherwise. Hence:

$$g(x_i, \rho) = 1 - \left[(1-\rho) + \rho \prod_{j=1}^{m} f(V_j)^{z_{ij}}\right] \tag{1}$$

The probability of not being vaccinated given that you are in the accessible population, $f(V_j)$ should be some function of the number of doese nominally distributed in campaign j, v_j, and the size of the accessible target population for that activity, ρN_j.

If all nominally distributed doses go into a unique vacinee in the target population, then $f(v_j, \rho N_j) = 1 - v_j/(\rho N_j)$. However, it seems we can assume that all nominally distributed doses do not result in a unique vaccinee within the target population. If we consider our doses to be a sequence, $k = 0 \ldots (v_j - 1)$, it further seems reasonable to assume that the chances of the first dose in this sequence is more likely to result in a unique vaccinee than later doses. This effect can be captured by the equation:

$$f(v_j, \rho N_j) = \prod_{k=0}^{v_j-1} \left(1 - \frac{1}{\rho N_j - k(1-\psi)}\right) \tag{2}$$

where ψ is a discount factor on how much the effective denominator changes on additional doses. That is, the term $-k(1-\psi)$ denotes how much the effective denominator (i.e., the number of people competing for doses) decreases because k doses have been given. If a campaign is perfect, then $\psi = 0$, and each dose in the sequence decreases the denominator by exactly 1 (and $f(v_j, \rho N_j) = 1 - v_j/(\rho N_j)$). If a campaign is effectively at random (i.e., the fact that doses have been previously distributed does not increase a new person's chance of receiving the next dose) then $\psi = 1$, and the probability of receiving (or avoiding) a dose remains constant. We would expect most vaccination activities to fall somewhere in this range. However, while it may be unlikely, we can even imagine a situation where there are "vaccine hungry" individuals who try to get vaccinated as many times as possible. In this case $\psi > 1$, and subsequent doses are even less likely to result in a unique vaccinee. Because values of ψ less than 0 are nonsensical (no dose can result in more than 1 vaccinee), we restate ψ in terms of α:

$$\psi = e^{\alpha}$$

We will use e^{α} instead of ψ throughout the supplement. Equation 1 now becomes:

$$g(x_i; \rho, \alpha) = 1 - \left[(1-\rho) + \rho \prod_{j=1}^{m} \left(\prod_{k=0}^{v_j-1} \left(1 - \frac{1}{\rho N_j - k(1-e^{\alpha})}\right)\right)^{z_{ij}}\right] \tag{3}$$

This equation can be further simplified by finding a closed form solution for the inner product as detailed in section 2 below.

In a cross sectional survey we observe a set of individuals with ages $\boldsymbol{x} = \{x_1, \cdots, x_n\}$ and corresponding vaccination statuses $\boldsymbol{y} = \{y_1, \cdots, y_n\}$, where $y_i = 1$ denotes having ever been vaccinated, and $y_i = 0$ denotes having never been vaccinated. If we assume all y_i are independent events, then the likelihood of observing the cross sectional data given ρ and α is:

$$L(\rho, \alpha; \boldsymbol{x}, \boldsymbol{y}) = \prod_{i=1}^{n} g(x_i; \rho, \alpha)^{y_i} (1 - g(x_i; \rho, \alpha))^{1-y_i} \tag{4}$$

2 Derivation of Simplified Form for Vaccination Probability

The probability that person i is vaccinated is:

$$g(x_i;\rho,\alpha) = 1 - \left[(1-\rho) + \rho\prod_{j=1}^{m}\left(\prod_{k=0}^{v_j-1}\left(1-\frac{1}{\rho N_j - k(1-e^{\alpha})}\right)\right)^{z_{ij}}\right] \tag{5}$$

$$= \rho - \rho\prod_{j=1}^{m}\left(\prod_{k=0}^{v_j-1}\left(1-\frac{1}{\rho N_j - k(1-e^{\alpha})}\right)\right)^{z_{ij}} \tag{6}$$

$$= \rho\left(1 - \prod_{j=1}^{m}\left(\prod_{k=0}^{v_j-1}\left(1-\frac{1}{\rho N_j - k(1-e^{\alpha})}\right)\right)^{z_{ij}}\right) \tag{7}$$

Let the portion of this equation that depends on v_j and ρN_j be designated $f(v_j, \rho N_j)$:

$$f(v_j, \rho N_j) = \prod_{k=0}^{v_j-1}\left(1-\frac{1}{\rho N_j - k(1-e^{\alpha})}\right) \tag{8}$$

Dropping the subscripts and taking $\rho N_j = N$ for convienence, note that:

$$\begin{aligned} f(v,N) &= \prod_{k=0}^{v-1}\left(1-\frac{1}{N - k(1-e^{\alpha})}\right) \\ &= \prod_{k=0}^{v-1}\frac{N - k(1-e^{\alpha}) - 1}{N - k(1-e^{\alpha})} \\ &= \prod_{k=0}^{v-1}\frac{1 - \frac{k}{N}(1-e^{\alpha}) - \frac{1}{N}}{1 - \frac{k}{N}(1-e^{\alpha})} \end{aligned}$$

Let $q = v/N$ and $a = (1-e^{\alpha})$:

$$
\begin{aligned}
f(v, N) &= f(qN, N) \\
&= \prod_{k=0}^{qN-1} \frac{1 - \frac{k}{N}a - \frac{1}{N}}{1 - \frac{k}{N}a} \\
&= \left(\prod_{k=0}^{qN-2} \frac{1 - \frac{k}{N}a - \frac{1}{N}}{1 - \frac{k}{N}a} \right) \left(\frac{1 - \frac{qN-1}{N}a - \frac{1}{N}}{1 - \frac{qN-1}{N}a} \right) \\
&= \left(\prod_{k=0}^{qN-2} \frac{1 - \frac{k}{N}a - \frac{1}{N}}{1 - \frac{k}{N}a} \right) \left(\frac{1 - qa + \frac{a}{N} - \frac{1}{N}}{1 - qa + \frac{a}{N}} \right) \\
&= \prod_{k=1}^{qN} \frac{1 - qa + \frac{ka}{N} - \frac{1}{N}}{1 - qa + \frac{ka}{N}}
\end{aligned}
$$

Hence:

$$
\begin{aligned}
\log f(qN, N) &= \sum_{k=1}^{qN} \log\left(1 - qa + \frac{ka}{N} - \frac{1}{N}\right) - \sum_{k=1}^{qN} \log\left(1 - qa + \frac{ka}{N}\right) \\
&= \frac{N}{a} \left[\frac{a}{N} \sum_{k=1}^{qN} \log\left(1 - qa + \frac{ka}{N} - \frac{1}{N}\right) - \frac{a}{N} \sum_{k=1}^{qN} \log\left(1 - qa + \frac{ka}{N}\right) \right]
\end{aligned}
$$

Hence, by the rectangular quadrature formula:

$$
\begin{aligned}
\log f(qN,N) \approx & \frac{N}{a}\left[\int_{1-qa+\frac{a}{N}-\frac{1}{N}}^{1-\frac{1}{N}} \log x dx - \int_{1-qa+\frac{a}{N}}^{1} \log x dx\right] \\
=& \frac{N}{a}\left[\left[x\log x - x\right]_{1-qa+\frac{a}{N}-\frac{1}{N}}^{1-\frac{1}{N}} - \left[x\log x - x\right]_{1-qa+\frac{a}{N}}^{1}\right] \\
=& \frac{N}{a}\left[\left(1-\frac{1}{N}\right)\log\left(1-\frac{1}{N}\right) - \left(1-\frac{1}{N}\right)\right. \\
& \quad - \left(1-qa+\frac{a}{N}-\frac{1}{N}\right)\log\left(1-qa+\frac{a}{N}-\frac{1}{N}\right) + \left(1-qa+\frac{a}{N}-\frac{1}{N}\right) \\
& \quad \left. -1\log 1 + 1 + \left(1-qa+\frac{a}{N}\right)\log\left(1-qa+\frac{a}{N}\right) - \left(1-qa+\frac{a}{N}\right)\right] \\
=& \frac{N}{a}\left[\left(1-\frac{1}{N}\right)\log\left(1-\frac{1}{N}\right) + \right. \\
& \quad - \left(1-qa+\frac{a}{N}-\frac{1}{N}\right)\log\left(1-qa+\frac{a}{N}-\frac{1}{N}\right) \\
& \quad \left. + \left(1-qa+\frac{a}{N}\right)\log\left(1-qa+\frac{a}{N}\right)\right] \\
=& \frac{N}{a}\log\left(1-\frac{1}{N}\right) - \frac{1}{a}\log\left(1-\frac{1}{N}\right) - \frac{N}{a}\log\left(1-qa+\frac{a}{N}-\frac{1}{N}\right) \\
& \quad + Nq\log\left(1-qa+\frac{a}{N}-\frac{1}{N}\right) - \log\left(1-qa+\frac{a}{N}-\frac{1}{N}\right) \\
& \quad + \frac{1}{a}\log\left(1-qa+\frac{a}{N}-\frac{1}{N}\right) + \frac{N}{a}\log\left(1-qa+\frac{a}{N}\right) \\
& \quad - Nq\log\left(1-qa+\frac{a}{N}\right) + \log\left(1-qa+\frac{a}{N}\right)
\end{aligned}
$$

Therefore (see limit calculations below):

$$\begin{aligned}
\lim_{N\to\infty} \log f(qN,N) &= \frac{1}{a}\lim_{N\to\infty} N\log\left(1-\frac{1}{N}\right) \\
&\quad - \left(\frac{1}{a}-q\right)\lim_{N\to\infty} N\log\left(1-qa+\frac{a}{N}-\frac{1}{N}\right) \\
&\quad + \frac{1}{a}\log\left(1-qa\right) \\
&\quad + \left(\frac{1}{a}-q\right)\lim_{N\to\infty} N\log\left(1-qa+\frac{a}{N}\right) \\
&= -\frac{1}{a} - \left(\frac{1}{a}-q\right)\frac{a-1}{1-qa} + \frac{1}{a}\log\left(1-qa\right) + \left(\frac{1}{a}-q\right)\frac{a}{1-qa} \\
&= \frac{1}{a}\log\left(1-qa\right)
\end{aligned}$$

Therefore:

$$\begin{aligned}
\lim_{N\to\infty} f(qN,N) &= \left(1-qa\right)^{1/a} \\
&= \left(1-q(1-e^{\alpha})\right)^{1/(1-e^{\alpha})}
\end{aligned}$$

Note that the above expression is undefined when $\alpha = 0$. However:

$$\lim_{a\to 0}\frac{log(1-qa)}{a} = \lim_{a\to 0}\frac{1}{1-qa}(-q) = -q$$

Therefore, for large N:

$$f(v,N) \approx \begin{cases} e^{-v/N} & \text{if } \alpha = 0 \\ \left(1-\frac{v}{N}(1-e^{\alpha})\right)^{1/(1-e^{\alpha})} & \text{otherwise} \end{cases} \tag{9}$$

And:

$$g(x_i;\rho,\alpha) \approx \begin{cases} \rho\left[1-\prod_{j=1}^{m}\left(e^{-v_j/\rho N_j}\right)^{z_{ij}}\right] & \text{if } \alpha = 0 \\ \rho\left[1-\prod_{j=1}^{m}\left(\left(1-\frac{v_j}{\rho N_j}(1-e^{\alpha})\right)^{1/(1-e^{\alpha})}\right)^{z_{ij}}\right] & \text{otherwise} \end{cases} \tag{10}$$

Note that this convergence appears to occur very quickly. Empirically, it appears that this value is accurate to three decimal places for $N > 100$ in sample scenarios.

LIMITS USING L'HOSPITAL RULE

$$\lim_{N\to\infty} N\log\left(1-\frac{1}{N}\right) = \lim_{x\to 0}\frac{(\log(1-x))'}{x'} = \lim_{x\to 0}\frac{1}{1-x}(-1) = -1$$

$$\lim_{N\to\infty} N\log\left(1-qa+\frac{a}{N}-\frac{1}{N}\right) = \lim_{x\to 0}\frac{(\log(1-qa+ax-x))'}{x'} = \lim_{x\to 0}\frac{1}{1-qa+ax-x}(a-1)$$
$$= \frac{a-1}{1-qa}$$

$$\lim_{N\to\infty} N\log\left(1-qa+\frac{a}{N}\right) = \lim_{x\to 0}\frac{\left(\log\left(1-qa+\frac{a}{N}\right)\right)'}{x'} = \lim_{x\to 0}\frac{1}{\left(1-qa+\frac{a}{N}\right)}(a)$$
$$= \frac{a}{1-qa}$$

3 Individual Campaign Coverage

Denote the actual coverage of a campaign j to be c_j. Note that c_j is the probability of a person covered only by campaign (or pseudo-campaign) j being vaccinated. Hence:

$$c_j = \begin{cases} \rho\left[1-e^{-v_j/\rho N_j}\right] & \text{if } \alpha = 0 \\ \rho\left[1-\left(1-\frac{v_j}{\rho N_j}(1-e^{\alpha})\right)^{1/(1-e^{\alpha})}\right] & \text{otherwise} \end{cases} \tag{11}$$

4 Routine Vaccination

Routine vaccination differs from campaigns in that children are vaccinated over a much larger time scale than is true of campaigns. However, routine vaccination can be modeled within our framework as a special type or vaccination activity.

Consider R years of routine vaccination activitiy, $1 \ldots R$. Denote the event of a member of the accessible population having the "opportunity" for vaccination during year j of routine vaccination as O_j and assume that each individual only has one routine vaccination opportunity. Further, assume that if the routine vaccination opportunity occurs during a given year then the probability of avoiding vaccination during that opportunity follows the same general form for activities:

$$\Pr(\text{not vaccinated by routine}|O_j) = f(v_j, \rho N_j)$$

If we let $\Pr(\bar{O})$ be the probability of having not yet had the opportunity for routine

vaccination, then:

$$\Pr(\text{not vaccinated by routine}) = \Pr(\bar{O}) + \sum_{j=1}^{R} f(v_j, \rho N_j) \Pr(O_j)$$

If we assume that each child has a probability $F_R(x)$ be the probability of having had your routine vaccination probability by age x. The the probability that person i is not vaccinated in a routine campaign is:

$$f_R(x_i, \boldsymbol{v}, \boldsymbol{N}) = (1 - F_R(x_i)) + \sum_{j=1}^{R} f(v_j, \rho N_j) \left(F_R(x_{ij} + l_j) - F_R(x_{ij})\right) \tag{12}$$

where x_{ij} is person i's age at the beginning of routine vaccination year j and l_j is the length of vaccination year j (12 months for all years except for the year the data was collected). In other words, routine vaccination becomes a pseudo-campaign representing the weighted sum of the coverage in all of the years of routine vaccination, where the weights represent the probability that routine vaccination happened in that year:

$$f_R(x_i, \boldsymbol{v}, \boldsymbol{N}) = w_i^* + \sum_{j=1}^{R} w_{ij} f(v_j, \rho N_j) \tag{13}$$

$$w_{ij} = F_R(x_{ij} + l_j) - F_R(x_{ij}) \tag{14}$$

$$w_i^* = 1 - F_R(x_i) \tag{15}$$

And the probability for vaccination for a given individual becomes:

$$g(x_i; \rho, \alpha) = \rho \left[1 - f_R(x_i, \boldsymbol{v}_R, \boldsymbol{N}_R) \prod_{j=1}^{m} f(v_j, \rho N_j)\right] \tag{16}$$

where m now represents the number of proper campaigns $\boldsymbol{v}_R$ and $\boldsymbol{N}_R$ are the number of doses distributed during routine vaccination activities.
